# Enhancing margarine oxidative stability, antioxidant retention, and sensory quality via tragacanth-chitosan hydrogel microencapsulation of supercritical CO₂-extracted green coffee

**DOI:** 10.1016/j.fochx.2025.102580

**Published:** 2025-05-23

**Authors:** Masoumeh Javadpour, Elahesadat Hosseini, Leila Nateghi, Sara Bazrafshan

**Affiliations:** a Department of Food Science and Technology, VaP.C, Islamic Azad University, Varamin, Iran; b Department of Chemical Engineering, Payame Noor University, Tehran, Iran; c Department of Food Science and Technology, Science and Research Branch, Islamic Azad University, Tehran, Iran

**Keywords:** Green coffee, Margarine, Microencapsulation, Oxidative stability, Tragacanth-chitosan hydrogel

## Abstract

Margarine's susceptibility to oxidative deterioration was addressed by microencapsulating supercritical CO₂-extracted green coffee antioxidants (GCSE) in tragacanth-chitosan (TG-CS) hydrogels. Optimized extraction (70 °C, 30 MPa, 20 % ethanol) yielded GCSE with high phenolic content (36.35 mg GAE/g dw) and antioxidant activity (89.76 % DPPH, 91.79 % ABTS inhibition). Encapsulation at a 1:3 TG-CS ratio achieved 85.28 % efficiency, retaining 149.54 mg GAE/100 g phenolics. Characterization (SEM, FTIR, XRD) confirmed micro-scale particles (0.928 μm) with moderate stability (Zeta potential: −17.66 mV) and sustained release (67.33 % over 6 h). In margarine, encapsulated GCSE (T2) maintained oxidative stability (peroxide: 2.02 meq/kg; p-anisidine: 4.88 mMol/kg; acid value: 1.81 mg NaOH/g), comparable to synthetic TBHQ (T3), while the control (T4) deteriorated rapidly. T2 retained 59.48 % DPPH activity (vs. 51.06 % for free GCSE, T1) and matched T3's sensory quality (aroma, texture, color; *p* > 0.05). TG-CS microencapsulation offers a dual-functional, clean-label solution for lipid-based foods, enhancing shelf life without compromising natural efficacy**.**

## Highlights

• Supercritical-CO₂ with ethanol co-solvent extracted antioxidants from green coffee.
• Bioactives were microencapsulated using chitosan-tragacanth hydrogel carriers.
• Encapsulation enhanced margarine's oxidative stability for up to 90 days.
• Antioxidant effect was comparable to TBHQ in margarine with encapsulated extract.
• Encapsulated extract maintained sensory characteristics comparable to TBHQ sample.

## 1 Introduction

Lipids play a pivotal role in human nutrition as a concentrated source of energy and a rich reservoir of essential fatty acids and fat-soluble vitamins. However, their inherent susceptibility to oxidation presents a significant challenge to maintaining the quality and safety of foods containing lipids. Lipid oxidation is recognized as the primary non-microbial mechanism that drives quality degradation and rancidity in edible oils, fats, and products like margarine, a widely used butter alternative made from vegetable oils. This process progresses through chain reactions involving free radicals or non-radical species and is exacerbated by environmental factors such as light, oxygen, and heat (Serra et al., 2024). The detrimental effects of lipid oxidation extend beyond sensory degradation, such as unpleasant odors and textural changes, to nutritional losses and the formation of potentially toxic compounds (Serra et al., 2024). Despite advancements in food preservation techniques, the use of antioxidants remains the most efficient, cost-effective, and practical approach. Antioxidants play a critical role in extending the shelf life of lipid-rich foods, preserving their nutritional integrity, and inhibiting the formation of harmful byproducts (Yousefi, Hosseini, et al., 2024). Both synthetic (e.g., butylated hydroxyanisole [BHA], butylated hydroxytoluene [BHT], propyl gallate [PG], and tert-butyl hydroquinone [TBHQ]) and natural antioxidants are effective in reducing lipid oxidation by donating electrons and quenching free radicals. Synthetic antioxidants, widely introduced in the early 20th century to retard lipid oxidation, have been extensively used in the food industry. However, concerns about their potential health risks, including toxicity and carcinogenic effects, have significantly shifted consumer and industry preferences toward safer, natural alternatives. This growing interest in natural antioxidants has highlighted plant-derived compounds like polyphenols (e.g., flavonoids, phenolic acids), and terpenoids for their strong antioxidant activity and safety. Polyphenols, in particular, stand out for their structural diversity and health benefits, offering robust protection against oxidative damage (Nateghi & Hosseini, 2023). Coffee is a globally significant product, with green coffee beans being rich in proteins, lipids, and bioactive compounds like caffeine and chlorogenic acid, which contribute to their high nutritional and functional value (Barajas-Álvarez et al., 2021). These beans contain essential fatty acids such as palmitic, oleic, and linoleic acids, and their antioxidant properties, primarily from chlorogenic acids, tannins, and lignans, provide health benefits. Chlorogenic acid regulates glucose metabolism, reduces oxidative stress, and lowers the risks of cancer and cardiovascular diseases (Barajas-Álvarez et al., 2021). Caffeine enhances alertness, reduces fatigue, and aids memory retention while alleviating Parkinson's disease symptoms (Ilgaz et al., 2018). Several techniques are commonly employed to extract valuable bioactive compounds from coffee, including pressing, Soxhlet extraction, and supercritical fluid extraction (SFE). Soxhlet extraction is widely utilized in laboratory research, whereas pressing is preferred for industrial-scale applications due to its simplicity and scalability (Ribeiro et al., 2024). Among these methods, SFE stands out as a sustainable and eco-friendly approach, offering significant advantages such as lower operational costs, reduced extraction times, and minimal environmental impact (Barajas-Álvarez et al., 2021). Operating under carefully controlled conditions, SFE effectively preserves heat- and light-sensitive compounds, making it an ideal choice for both laboratory and industrial purposes. The efficiency of SFE, especially when using CO_2_ as the extraction medium, is significantly enhanced by the addition of co-solvents like ethanol and methanol. This combination improves the extraction of bioactive compounds, with studies showing an increase in antioxidant capacity by up to 12.5 times during coffee oil extraction (Coelho et al., 2020). Similarly, the addition of ethanol in SFE under optimal conditions increased the extraction yield to 7.7 %, with enhanced antioxidant potential and high concentrations of caffeine and chlorogenic acids. By incorporating co-solvents, SFE not only boosts the antioxidant properties of the extracted compounds but also enhances the overall yield and efficiency of bioactive extractions (Barajas-Álvarez et al., 2021). The instability of bioactive compounds under conditions such as light, temperature, oxygen, and digestion necessitate advanced protective measures to preserve their integrity. Encapsulation has emerged as a reliable strategy to prevent degradation, enhance bioavailability, and mask undesirable tastes and odors, ensuring the stability and functionality of these sensitive compounds. Beyond protection, microencapsulation offers several advantages, including controlled release, improved ease of handling, uniform dispersion, and prolonged nutrient retention during processing and storage. This process leverages stable polymer networks to encapsulate hydrophobic macromolecules and antioxidants like chlorogenic acids, thereby enhancing their resistance to oxidative and thermal degradation, extending shelf life, and maintaining bioactivity (Mu et al., 2024). Proteins, polysaccharides, and gums play a critical role as carriers and emulsifiers in encapsulation, contributing to structural stability and functional performance (Yousefi, Nateghi, & Rashidi, 2024). Among these, polysaccharide-based hydrogels such as chitosan (CS) stand out for their cost-effectiveness, versatility, and ability to shield bioactive compounds from environmental factors, including variations in pH and temperature. CS-based hydrogels also facilitate controlled release, enhancing sensory qualities and consumer appeal (Najafi-Soulari et al., 2016). However, the inherent pH sensitivity and mechanical limitations of CS necessitate improvements through blending with complementary polymers. Tragacanth gum (TG), recognized for its stability, biocompatibility, and emulsifying properties, is an excellent complement to CS. Its ability to enhance structural integrity and functional versatility makes it a promising biopolymer for encapsulation applications (Potaś et al., 2020). To our knowledge, no prior research has explored the application of a TG-CS hydrogel for the microencapsulation of green coffee supercritical extract (GCSE) in margarine formulations. This innovative approach was designed to enhance oxidative stability and sensory attributes while offering a natural antioxidant solution. In this study, TG-CS hydrogels were developed as a delivery system, incorporating green coffee extract obtained through supercritical fluid extraction. The extraction process was optimized under controlled conditions (40–70 °C, 200–300 MPa, with a constant 20 % co-solvent) to maximize the yield of phenolic compounds, assessed through extraction yield (EY), total phenolic content (TPC), and antioxidant activities (DPPH and ABTS). Once the optimal extraction conditions were established, TG-CS hydrogels were formulated to encapsulate the optimized GCSE, and encapsulation efficiency (EE) and TPC were determined. The optimized formulation underwent comprehensive characterization, including particle size analysis, SEM, FTIR, and XRD, and an evaluation of its gastrointestinal stability. In the final stage of the study, the optimized microencapsulated GCSE was incorporated into margarine, and its oxidative stability, antioxidant capacity, and sensory attributes were evaluated during 90 days of refrigerated storage.

## 2 Materials and methods

### 2.1 Materials

Green arabica coffee beans were harvested in Ethiopia between September and December 2023. Following harvest, the beans underwent processing, where the outer skin was removed, and fermentation was carried out to break down the mucilage. They were then thoroughly washed and dried at a controlled temperature of 50 °C. The beans' proximate composition was found to be 12.47 % fat, 7.60 % carbohydrates, 49.08 % moisture, 4.00 % ash, and 8.53 % protein. On average, green coffee beans contain approximately 11.65 mg/g of caffeine and 35.62 mg/g of 5-caffeoylquinic acid (Mengesha et al., 2024). TG, mainly composed of galacturonic acid, galactose, rhamnose, arabinose, mannose, glucose, and xylose obtained from local shop, and medium molecular weight CS (MW: 190–310 kDa, deacetylation degree: 75–80 %) from Sigma-Aldrich (USA) were used in this study. Folin-Ciocalteu reagent, DPPH, ABTS^●+^, and gallic acid were also obtained from Sigma-Aldrich (USA). Palm stearin, palm oil, and palm kernel oil were supplied by Adonis Gol Daru Company (Iran), while soy lecithin was obtained from Gujarat (China). Canola oil and hydrogenated sunflower oil were purchased from Behshahr Industrial Company (Iran). All other analytical-grade chemicals used in the study were procured from Merck (Germany).

### 2.2 Extraction of green coffee extract using SFE

For the extraction process, dried green coffee beans were first ground using a disc mill (Conil, TRANQULIO, Spain) and passed through a 30-mesh sieve. The extraction procedure was adapted from Barajas-Álvarez et al. (2021) with modifications, utilizing the multifunctional Suprex MPS/225 system. Precisely 21 g of ground coffee were weighed and placed into the extraction vessel. To initiate the SFE, the extraction vessel was first pressurized to the target pressure (20–30 MPa) and heated to the desired temperature (40–70 °C), to achieve the supercritical state (Table 1). Once the conditions were stabilized, carbon dioxide (CO₂) along with a 20 % ethanol solution as a co-solvent was pumped into the extraction vessel at a constant total flow rate of 10 g/min for 180 min. Following extraction, the solvent was removed using a vacuum evaporator (TAM, Iran) at 50 °C. The obtained extract was protected from light by wrapping it in aluminum foil and subsequently stored at 4 °C for further analysis.

**Table 1:** Extraction yield (EY), total phenolic content (TPC), and antioxidant activity for different test conditions of GCSE.

Test No.	T$\;\left({}^{{}^{\circ}}C\right)$	P (Mpa)	Co (%)	EY⁎ (%)	TPC⁎ (mg GAE/g dw)	Antioxidant Activity⁎	
						DPPH (%)	ABTS^●+^ (%)
GCSE_1_	40	20	20	5.403 ^f^ ± 0.398	26.046 ^e^ ± 0.350	76.600 ^e^ ± 0.254	78.431 ^e^ ± 0.222
GCSE _2_	50	20	20	6.776 ^e^ ± 0.433	27.088 ^de^ ± 0.450	79.283 ^d^ ± 0.936	80.613 ^d^ ± 0.847
GCSE _3_	60	20	20	7.582 ^e^ ± 0.232	27.413 ^d^ ± 617	80.757 ^cd^ ± 0.601	82.168 ^c^ ± 0.485
GCSE _4_	70	20	20	8.819 ^d^ ± 0.374	27.577 ^d^ ± 0.241	81.044 ^c^ ± 0.645	82.288 ^c^ ± 0.541
GCSE _5_	40	30	20	10.141 ^c^ ± 0.423	31.788 ^c^ ± 0.158	85.767 ^b^ ± 0.548	87.381 ^b^ ± 0.515
GCSE _6_	50	30	20	10.741 ^c^ ± 0.136	32.403 ^c^ ± 0.325	87.195 ^b^ ± 0.087	88.414 ^b^ ± 0.183
GCSE _7_	60	30	20	12.405 ^b^ ± 0.374	34.645 ^b^ ± 0.435	89.580 ^a^ ± 0.456	91.400 ^a^ ± 0.551
GCSE _8_	70	30	20	14.937 ^a^ ± 0.281	36.350 ^a^ ± 0.277	89.762 ^a^ ± 0.471	91.792 ^a^ ± 462
*P*-value				0.000	0.000	0.000	0.000
F-value				244.60	314.5	241.02	294.65

⁎ Mean ± SD, *n* = 3. Different letters (a-e) within each column indicate significant differences (*p ≤ 0.05*). Temperature (T), Pressure (P), Co-solvent content (Co). Note: EY (%) refers to the extraction yield calculated based on the initial sample weight of 21 g.

### 2.3 Physicochemical characteristics of GCSE

#### 2.3.1 Extraction yield (EY) determination

The extraction yield was calculated as the percentage of the recovered GCSE mass to the initial coffee bean mass (Barajas-Álvarez et al., 2021).

#### 2.3.2 Determination of Total phenolic content (TPC)

The TPC was determined using the Folin-Ciocalteu method, following the procedure described by Jeszka-Skowron et al. (2016), with gallic acid as the reference standard (R^2^ = 0.995). For the analysis, 30 mg of GCSE was dissolved in 2 mL of ethanol, and a 30 μL sample was mixed with 150 μL of diluted Folin-Ciocalteu reagent. After 4 min, 120 μL of 0.71 M sodium carbonate was added, and the mixture was incubated in the dark at 20 °C for 1 h. Finally, absorbance was recorded at 765 nm using a UV–Vis spectrophotometer (UV 2100, UNICO, China). TPC was expressed as milligrams of gallic acid equivalents (GAE) per gram of dry weight (dw) of the extract.

#### 2.3.3 Antioxidant activity assessments

##### 2.3.3.1 DPPH activity

The DPPH (2,2-diphenyl-1-picrylhydrazyl) radical scavenging activity was assessed based on the method outlined by Jeszka-Skowron et al. (2016). In summary, GCSEs were dissolved in ethanol at a concentration of 15 mg/mL. A 20 μL aliquot of the sample was then combined with 200 μL of a 500 μM ethanolic DPPH solution. The mixture was incubated in the dark at room temperature for 30 min, after which the absorbance was measured spectrophotometrically at 516 nm against a blank. The scavenging activity was calculated as a percentage using the following Eq. (1).

(1) $$\%\text{DPPH scavenging capacity}=\left(A_{516\;\text{control}}-A_{516\;\text{sample}}/A_{516\;\text{control}}\right)\times 100$$

where A _control_ and A _sample_ represent the absorbance of DPPH in pure ethanol and DPPH in the presence of the GCSE extract, respectively.

##### 2.3.3.2 ABTS^●+^ activity

The total antioxidant activity of the GCSE extract was assessed using the ABTS (2,2′-azino-bis(3-ethylbenzothiazoline-6-sulphonic acid)) radical cation assay, following Jeszka-Skowron et al. (2016) without conversion to Trolox equivalents. GCSE (15 mg/mL) was dissolved in ethanol, and a 20 μL sample was mixed with 200 μL of ABTS^●+^ solution (prepared 24 h earlier with 7 mM ABTS and 2.45 mM potassium persulfate). The solution was diluted to an absorbance of 0.70 ± 0.02 at 734 nm, incubated in the dark for 6 min, and its absorbance was measured spectrophotometrically at 734 nm. The antioxidant activity was calculated by using the following Eq. (2):

(2) $$\text{ABTS scavenging capacity}\%=\left(A_{734\;\text{control}}-A_{734\;\text{sample}}/A_{734\;\text{control}}\right)\times 100$$

Where A _control_ and A _sample_ are the absorbance of the ABTS solution with ethanol and GCSE extract, respectively.

### 2.4 Fatty acid profile of selected GCSE

The fatty acid profile of the selected GCSE, including linoleic, myristic, palmitic, oleic, stearic, linolenic, arachidonic, behenic, and lignoceric acids, was analyzed using gas chromatography-flame ionization detection (GC-FID). The procedure followed the methodology outlined by Granados-Vallejo and Processes (2019). For the preparation of fatty acid methyl esters (FAMEs), the saponification step was carried out using a 0.5 N potassium hydroxide solution, while boron trifluoride-methanol was utilized as the esterification reagent. The separation and identification of fatty acids were conducted using a GC 7820 system (Agilent Technologies Inc., Palo Alto, CA, USA), equipped with a DB23 capillary column (60 m × 0.25 mm i.d × 0.25 μm film thickness of stationary phase). The quantification of indi*v*idual fatty acids was performed using external calibration standards.

### 2.5 Microencapsulation of selected GCSE in TG-CS hydrogel carrier

To prepare 1 wt% TG dispersions, 1 g of TG was dispersed in 90 mL of deionized water at 80 °C. The selected GCSE extract (0.5–1.5 % *w*/*v*) was then gradually incorporated into the TG dispersion while being homogenized for 15 min using an Ultraturrax homogenizer (IKA T 25, Deutschland, Germany). The mixtures were left to hydrate overnight to ensure complete swelling of TG. After hydration, the dispersions were washed with acetate buffer (pH 5.5) and allowed to air-dry at room temperature for 24 h. To prepare TG-CS hydrogel beads, the pre-prepared TG solution was added to a CS solution (1 % in 0.1 M acetic acid) at different concentrations (1, 2, and 3 wt%). The mixture was stirred overnight and then incubated for 30 min to facilitate gel formation. Then the hydrogels were centrifuged at 1000 rpm for 1 min at room temperature (approximately 25 °C) to eliminate any trapped air bubbles (Table 2) (Najafi-Soulari et al., 2016).

**Table 2:** Effect of optimized GCSE_8_ concentration and TG-CS ratio on EE and TPC.

Test No.	GCSE_8_⁎⁎ (%)	TG- CS ratio (%)	EE ⁎ (%)	TPC ⁎(mg/GAE/100 g)
M_1_	0.5	1:1	63.587 ^i^ ± 0.331	111.644 ^i^ ± 0.881
M_2_	0.5	1:2	66.010 ^h^ ± 0.790	116.053 ^h^ ± 1.689
M_3_	0.5	1:3	68.839 ^g^ ± 0.209	121.097 ^g^ ± 0.430
M_4_	1	1:1	80.525 ^c^ ± 0.540	141.427 ^c^ ± 1.195
M_5_	1	1:2	83.515 ^b^ ± 0.480	146.616 ^b^ ± 0.885
M_6_	1	1:3	85.284 ^a^ ± 0.415	149.542 ^a^ ± 0.881
M_7_	1.5	1:1	71.782 ^f^ ± 0.155	125.956 ^f^ ± 0.433
M_8_	1.5	1:2	75.419 ^e^ ± 0.537	130.775 ^e^ ± 1.158
M_9_	1.5	1:3	76.947 ^d^ ± 0.366	135.022 ^d^ ± 0.760
P-value			0.000	0.000
F-value			827.67	543.59

⁎ Mean ± SD, n = 3. Different letters (a-e) within each column indicate significant differences (*p ≤ 0.05*).

⁎⁎ Optimized GCSE (30 MPa, 70 °C, and 20 % co-solvent), Total phenolic content (TPC), Encapsulation efficiency (EE). Tragacanth gum (TG), as chitosan (CS).

### 2.6 Selection of optimized formulation

To determine the optimal formulation, the encapsulated samples were evaluated based on encapsulation efficiency (EE) and TPC. The formulation with the highest EE and TPC was selected for further characterization and advanced analyses.

#### 2.6.1 Encapsulation efficiency (EE) assessment

The phenolic content of the capsules was assessed using the Folin-Ciocalteu reagent, following the methodology outlined by da Silva Júnior et al. (2023) and calculated using the following Eq. 3.

(3) $$\mathrm{EE}\;\left(\%\right)=\left(\mathrm{TPC}-\mathrm{SPC}/\mathrm{TPC}\right)\times 100$$

Where: TPC = Total phenolic content used for preparation, SPC = Phenolic content that could not be encapsulated.

#### 2.6.2 TPC determination

The TPC of the encapsulated samples was determined based on the Folin-Ciocalteu method, following the approach described by Jeszka-Skowron et al. (2016). The results were reported as mg gallic acid equivalent per gram of dry matter (mg GAE/g DM).

### 2.7 Characteristics of optimized formulation

#### 2.7.1 Mean particle size and zeta potential

The physical properties of the optimized samples, including mean particle size and zeta potential, were assessed using dynamic light scattering (DLS) with a Zetasizer instrument (NanoSiz Malvern, Nano ZS 3600, UK). The optimized formulation was diluted with ultrapure water (1:10) and introduced into a specialized DLS measurement cell with a 1 cm path width. Measurements were performed at room temperature with a scattering angle of 173°. Additionally, the zeta potential was determined after appropriate dilution with distilled water.

#### 2.7.2 Scanning Electron microscopy (SEM)

The surface morphology and microstructure of the GCSE microcapsules were analyzed using an SEM (PS-230, Pmetron Co., Korea). To enhance conductivity, the samples were coated with a thin layer of gold using a sputter coater (BAL-TEC AG, Balzers, Liechtenstein) before imaging. The SEM analysis was conducted at an accelerating voltage of 10 kV with a magnification of 1000 × .

#### 2.7.3 Fourier-transform infrared spectroscopy (FTIR)

A FTIR spectrometer (Bruker, Vertex 70, Germany) was used to examine chemical bonds and identify functional groups. The spectra were obtained within the 400–4000 cm^−1^ wavenumber range, using 32 scans at a resolution of 4 cm^−1^ and a scan velocity of 1.6 kH. The KBr pellet technique was applied with a sample-to-KBr ratio of 1:100.

#### 2.7.4 X-ray diffraction (XRD)

The crystalline structure of the samples was analyzed using a Bruker D8 Advance X-ray diffractometer with Cu Kα radiation (λ = 0.154 nm), operated at 40 kV and 40 mA. Diffraction patterns were recorded over a 2θ range of 5° to 55° at a scanning speed of 10°/min to assess the structural properties of the samples.

#### 2.7.5 In vitro gastrointestinal digestion

The release rate of the optimized formulation was evaluated under simulated gastric and intestinal conditions using a modified dialysis method. In this regard, simulated gastric fluid (SGF, pH 4) was prepared with 2 mg/mL NaCl, 3 mg/mL pepsin, and 2 mg/mL of an additional component, while simulated intestinal fluid (SIF, pH 8.7) contained 8.6 mg/mL NaCl, 8.6 mg/mL KH₂PO₄, 2 mg/mL pancreatin, and 5 mg/mL bile salts. To initiate the study, 3 mL of the hydrogel sample was mixed with 3 mL of SGF, placed in a dialysis membrane bag with a molecular weight cutoff of 8.14 kDa, and immersed in 61 mL of SGF release medium for 120 min. Subsequently, 6 mL of SIF was added to the dialysis bag, which was then transferred to 121 mL of SIF release medium for 240 min. The experiment was conducted in an incubator maintained at 37 °C with continuous shaking at 121 rpm. At predetermined time intervals, 1 mL of the release medium was withdrawn and replaced with fresh medium. The collected samples were then centrifuged (Eppendorf, Germany) at 1000 g for 10 min, filtered through a 1.45 μm filter, and analyzed for hydrogel release using HPLC (Agilent, USA). A cumulative release profile of GCSE over 360 min was plotted based on Eq. (4):

(4) $$\text{Cumulative release}\;\left(\%\right)=\frac{Q_{1}}{Q_{0}}$$

Where Q_1_ represents the amount of encapsulated GCSE released at a given time (t), while Q_0_ refers to the initial quantity of GCSE within the capsules.

### 2.8 Margarine production

Margarine was prepared based on the method of Yousefi, Nateghi, and Rashidi (2024), the process involved blending an 80 % (*w*/w) lipid phase with a 20 % (w/w) aqueous phase using a blender. The lipid phase primarily consisted of 79.3 % canola oil and hydrogenated sunflower oil, along with 0.7 % of a mixed emulsifier containing glyceryl monostearate, glyceryl distearate, and lecithin. The aqueous phase included 1 % (w/w) sodium chloride to enhance the formulation. To enrich the margarine formulation, 100 ppm of optimized free GCSE (T1), 100 ppm of optimized bio-hydrogels encapsulating GCSE (T2), and 75 ppm of the synthetic antioxidant TBHQ (T3) were incorporated. The prepared margarine samples were then transferred to plastic containers and stored at 4 °C. To assess product stability and quality over time, antioxidant activity, oxidation stability, and sensory attributes were evaluated at 30th-day intervals throughout a 90th-day refrigerated storage period. A control sample (T4), consisting of margarine without any additives, was included for comparison.

#### 2.8.1 Margarine oxidative stability during cold storage

Oxidative stability was evaluated throughout storage by measuring the acid value (AV), peroxide value (PV), and p-anisidine value (p-AV) according to (ISO 3960, 2017; ISO 660, 2020; ISO 6885, 2016), respectively.

#### 2.8.2 DPPH assessment of margarine during cold storage

The antioxidant capacity of margarine samples containing both encapsulated and free antioxidants was assessed using the DPPH assay, following the method described by Yousefi, Hosseini, et al. (2024).

#### 2.8.3 Sensory evaluation of margarine during cold storage

The sensory properties, including aroma, taste, texture, and overall acceptability, were evaluated by a panel of 11 trained panelists aged 25 to 35 years. Each sample was provided in individual plastic containers, labeled with a three-digit code, and tested randomly by the panel. A five-point hedonic scale (1: dislike extremely; 5: like exceptionally) was used for scoring. Prior to the evaluation, all participants provided informed consent to participate in the study. As the samples were food-grade and safe for consumption, no formal ethical approval was required. The study followed ethical research guidelines, ensuring voluntary participation and confidentiality of responses.

### 2.9 Statistical analysis

To determine statistical significance, one-way ANOVA was performed, followed by Duncan's multiple range test at a 95 % confidence level (*p ≤ 0.05*). All experiments were conducted in triplicate, and the results were expressed as mean values with standard deviation. Statistical analysis was carried out using Minitab version 16.1.1.0 and the graphs were plotted using Excel 2013.

## 3 Results and discussion

### 3.1 Impact of extraction conditions on GCSE properties

Table 1 summarizes the effects of pressure, temperature, and co-solvent concentration on EY, TPC, and antioxidant activity of GCSE. As shown in Table 1, the EY varied from 5.403 ± 0.398 % to 14.937 ± 0.281 %, with the highest yield observed for GCSE_8_ under optimized conditions of 30 MPa pressure, 70 °C temperature, and 20 % ethanol as co-solvent for 180 min (*p ≤ 0.05*).

These findings are consistent with the results reported by Pokrovskiy et al. (2018), who demonstrated that increasing pressure from 20 MPa to 50 MPa while maintaining a constant temperature of 80 °C enhanced oil EY from 6 % to 17 %. Comparable trends have also been reported by Barajas-Álvarez et al. (2021), who investigated green coffee bean extraction under similar experimental conditions. Their study demonstrated that increasing the ethanol concentration as a co-solvent from 5 % to 20 % at 30 MPa nearly doubled the EY. Specifically, the use of pure supercritical CO₂ at 22.5 MPa and 60 °C resulted in a low EY of 0.9 %, whereas optimal conditions (20 % ethanol, 30 MPa, and 50 °C) yielded 7.68 %. This improvement was attributed to the increased density of the supercritical fluid and the enhanced solubilizing power of CO₂ in the presence of ethanol, which facilitated more efficient extraction. At higher pressures, the solvent density improves, boosting solubility and mass transfer. Conversely, the low EY at 22.5 MPa and 60 °C was likely due to the inadequate solvent density and the non-polar nature of pure CO₂, which limits its ability to dissolve polar or semi-polar compounds. The absence of ethanol in this setup significantly reduced extraction efficiency (Barajas-Álvarez et al., 2021).

Similarly, as shown in Table 1, TPC content increased with pressure up to 30 MPa and temperature up to 70 °C, ranging from 26.046 ± 0.350 to 36.350 ± 0.277 mg GAE/g dw. The greatest enhancement was observed at 30 MPa and 70 °C (GCSE_8_), highlighting the critical role of these parameters in optimizing extraction (*p ≤ 0.05*). This trend aligns with previous findings that supercritical fluid extraction at elevated pressure and temperature improves TPC yield. According to Barajas-Álvarez et al. (2021), the model-predicted optimal conditions for maximizing TPC were 5.38 mg GAE/g GCSE, achieved with 20 % co-solvent, 30 MPa pressure, and 62 °C temperature. Studies also indicate that combining supercritical CO₂ with ethanol as a binary solvent system consistently yields higher phenolic content compared to single-solvent systems (Romano et al., 2021). The addition of ethanol modifies the system's critical point by increasing both temperature and pressure, thereby enhancing the solvent's ability to dissolve highly polar compounds present in the sample.

### 3.2 Antioxidant activity of GCSE

This study evaluated the antioxidant activity of GCSE extracted using supercritical CO₂ (scCO₂) under different pressure and temperature conditions. The radical scavenging potential was determined using DPPH and ABTS^●+^ assays, with ANOVA confirming that extraction parameters significantly influenced antioxidant capacity (*p ≤ 0.05*). Increasing pressure from 20 to 30 MPa and temperature from 40 to 70 °C enhanced free radical inhibition (Table 1).

The treatment No.8 (GCSE_8_), extracted at the highest pressure and temperature, exhibited the most potent antioxidant activity (*p* *≤ 0.05*). A strong correlation between the DPPH and ABTS^●+^ methods suggests that similar antioxidant compounds in GCSE contribute to scavenging both radicals. Additionally, the positive correlation between TPC and free radical scavenging ability supports the role of polyphenols in enhancing antioxidant potency. Comparisons with previous research further highlight the influence of extraction conditions. Coelho et al. (2020) demonstrated that the addition of co-solvents like ethanol to scCO₂ significantly enhances DPPH inhibition, achieving values as high as 12.39 mg/mL at 30 MPa and 60 °C. Moreover, Marcucci et al. (2017) reported ABTS^●+^ values ranging from 20.39 to 37.02 g Trolox/100 g in coffee extracts, demonstrating how extraction technique and raw material influence bioactivity. These findings underscore the crucial role of extraction parameters in optimizing bioactive compound recovery. Among all samples, GCSE_8_ (70 °C / 30 MPa) was identified as the optimal extract, exhibiting the highest TPC, EY, and antioxidant activity.

### 3.3 Fatty acid composition of the optimized extract (GCSE_8_)

**Fig. 1:**
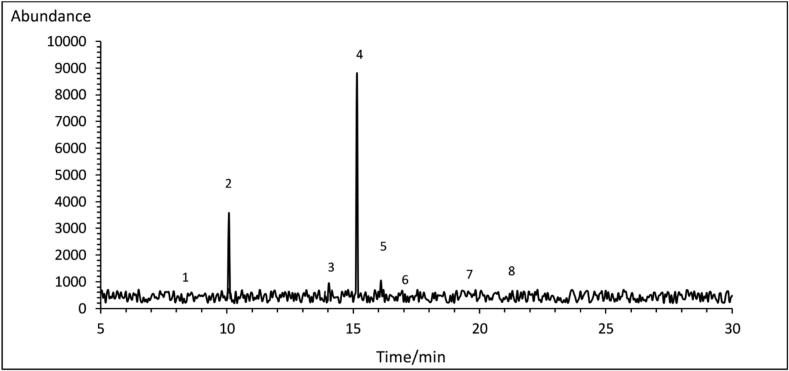
GC-FID chromatogram of fatty acids in optimized extract (GCSE_8_).

The chromatographic profile of fatty acids identified in the optimized GCSE through GC-FID is illustrated in Fig. 1, while their corresponding percentages are detailed in Table 1S. The results indicate that the optimized GCSE predominantly contained unsaturated fatty acids (UFAs), accounting for approximately 63.04 %. Among these, linoleic acid was the most abundant, comprising 50.239 ± 0.39 % of the total fatty acid content (Fig. 1, peak No.1). Regarding saturated fatty acids, palmitic acid (peak No.2) was present in the highest amount (22.499 ± 0.44 %), while stearic (peak No.5), oleic (peak No.3), Lignoceric (peak No.8), Behenic (peak No. 7), myristic (peak No,1) and arachidic (peak No.6) acids were detected in smaller proportions respectively (Fig. 1). However, some studies have reported palmitic acid as the most abundant fatty acid, reaching up to 46.10 % (Hurtado-Benavides et al., 2016). These discrepancies are likely attributed to variations in source, geographical origin, and extraction methods of coffee beans. Notably, the application of SFE does not seem to cause significant alterations in the fatty acid profile (Barajas-Álvarez et al., 2021).

### 3.4 Impact of microencapsulation on optimized GCSE_8_ characteristics

For microencapsulation, optimized GCSE_8_ was integrated into a TG-CS hydrogel carrier, testing various extract concentrations and TG-CS ratios.

As shown in Table 2, microencapsulation significantly affected both EE and TPC (*p ≤ 0.05*). Increasing the TG-CS ratio up to 1:3 enhanced the stability of the encapsulation matrix and improved the retention of bioactive compounds. The highest EE (85.284 ± 0.415 %) and TPC (149.542 ± 0.881 mg GAE/100 g) were achieved at an extract concentration of 1 % and a TG-CS ratio of 1:3. Although a further increase in extract concentration to 1.5 % provided more phenolic compounds, both EE and TPC decreased slightly. This reduction may be attributed to increased viscosity or matrix saturation, which could hinder the efficient encapsulation of bioactive compounds. These results suggest that beyond a certain concentration; the encapsulation matrix may become less effective due to physical limitations. The observed trends are consistent with a previous study by Jayanudin, and M., Wirawan, S. K., and Rochmadi. (2019) which reported that increasing the concentration of microencapsulating carriers such as chitosan improves EE by reinforcing matrix stability. Based on these findings, the M6 formulation was selected for further experiments due to its superior encapsulation performance and phenolic content retention.

### 3.5 Characteristics of the optimized microencapsulated GCSE in TG-CS hydrogel systems

#### 3.5.1 Mean particle size and zeta potential

Mean particle size and zeta potential are key parameters influencing the behavior of colloidal systems, particularly in encapsulation processes. These factors play a crucial role in determining stability and the interaction of bioactive compounds with the carrier. In Fig. 1S (a and b), the mean particle size and zeta potential values of the optimized microencapsulated sample (M6) are presented.

The results show that the mean particle size is 928 nm (0.928 μm), with a zeta potential of −17.66 mV. While the size is expressed in nanometers, it is relatively large and falls within the lower micro-scale range rather than the typical nanoparticle classification. Research on plant extract encapsulation using chitosan as a carrier has yielded diverse findings (Karimi et al., 2024; Khosravi et al., 2025). For example, Mu et al. (2024) produced microencapsulated green coffee oil powders using a combination of protein-polysaccharide complex coacervation and freeze drying, with particle sizes ranging from 72.57 to 295.00 μm, confirming the formation of micro-scale capsules. In contrast, Veiga et al. (2016) encapsulated roasted coffee oil using enzymatically crosslinked gelatin and gum Arabic, obtaining microcapsules with an average diameter of 29.30 ± 1.28 μm. These discrepancies highlight the influence of physicochemical properties, encapsulation techniques, and ingredient interactions on final particle size. Furthermore, the present study demonstrated a significant correlation between the zeta potential of microencapsulated GCSE and the stability of the microparticle suspension. The optimized formulation (M6), which had the highest TG-CS ratio (1:3), exhibited a negative zeta potential of −17.66 mV. Increasing CS concentration resulted in a less negative surface charge, facilitating stronger interactions between TG and CS due to chitosan's amino groups. A zeta potential beyond +30 mV or below −30 mV indicates enhanced stability, as stronger repulsive forces between particles effectively prevent aggregation. This highlights the crucial role of the CS coating in maintaining particle dispersion (Karimi et al., 2024).

#### 3.5.2 SEM

**Fig. 2:**
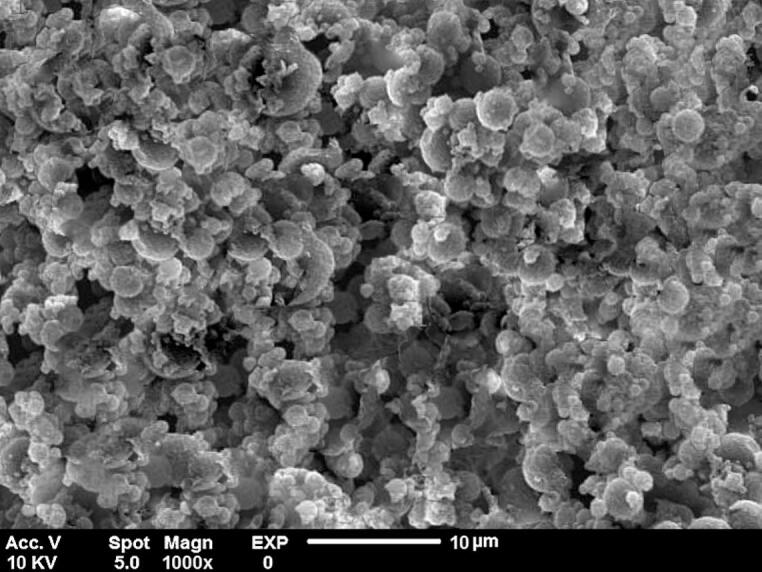
SEM micrograph at 1000× magnification for Optimized Microencapsulated Sample (M6).

The SEM image of the optimized microencapsulated sample (M6) reveals microparticles with an irregular, porous morphology (Fig. 2). This structural characteristic is likely a result of the microencapsulation process. The presence of agglomerates in certain regions suggests electrostatic interactions between the positively charged CS and negatively charged TG (Shirzadian et al., 2021). The relatively high chitosan content in the sample (TG-CS ratio of 1:3) may have contributed to its morphology, leading to the formation of microparticles with distinct structural features. Additionally, a higher degree of deacetylation (DD) in chitosan may also enhance the tendency for particle aggregation (Huang et al., 2015). The scale bar of 10 μm indicates that the microparticles are very small but still cannot be classified as nanoparticles. Therefore, we consider them as small micro-scale particles, which is consistent with the results obtained from DLS measurements. These findings suggest that the structural characteristics of the microcapsules may play a role in their stability and encapsulation efficiency.

#### 3.5.3 FTIR

**Fig. 3:**
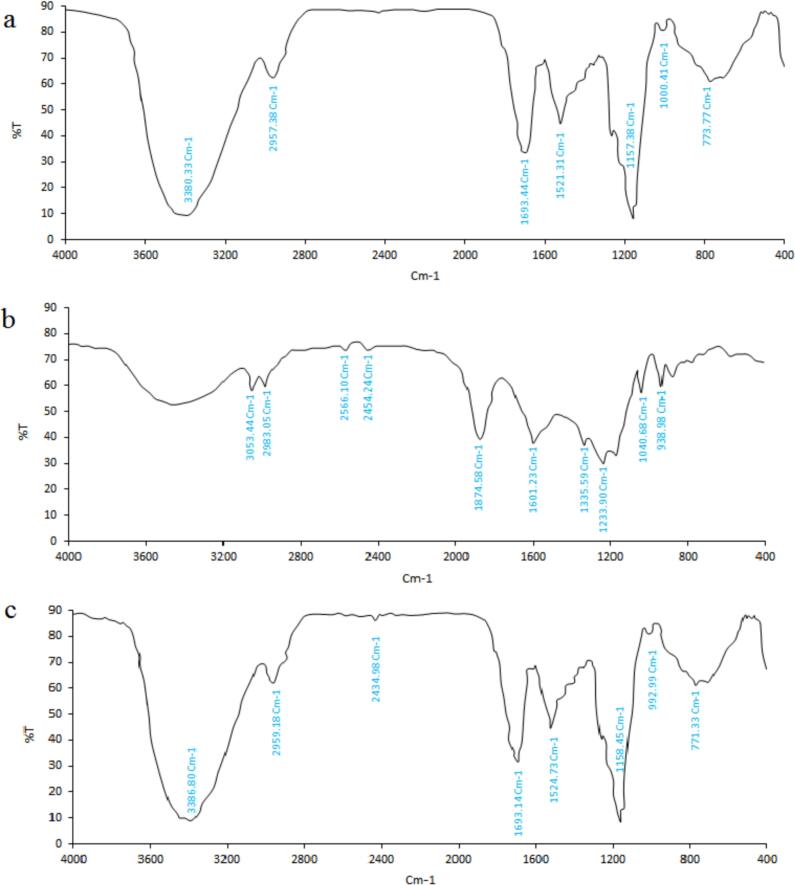
FTIR spectra of (a) TG-CS, (b) Optimized GCSE_8_, and (c) the Optimized Microencapsulated (M6).

FTIR analysis was performed to examine the chemical bonding and structural properties of the microparticles and their coating materials. Fig. 3 shows the FTIR spectra for the TG-CS carrier (a), optimized GCSE sample (b), and the optimized microencapsulated formulation (c). For the TG-CS carrier, distinct absorption bands indicate the presence of polysaccharide structures (Fig. 3a). A broad peak at 3398.13 ${\mathrm{cm}}^{-1}$ reflects O—H and N—H bond stretching, suggesting hydroxyl and amine groups (Potaś et al., 2020). The TG component shows O—H stretching between 3200 and 3500 ${\mathrm{cm}}^{-1}$, highlighting its hydrophilic nature, while the CS component displays a peak at 3455 ${\mathrm{cm}}^{-1}$, consistent with the TG-CS ratio (1:3). Additionally, peaks at 2927.38 ${\mathrm{cm}}^{-1}$ and 1593.44$\;{\mathrm{cm}}^{-1}$ correspond to C—H stretching in alkyl groups and N—H bending in amide (II) groups. The presence of amide I (C=O stretching) and amide II (N—H bending) bands at approximately 1655 ${\mathrm{cm}}^{-1}$ and 1559 ${\mathrm{cm}}^{-1}\;$further confirms the presence of amide groups in CS (Pokhrel et al., 2016). Absorption bands at 1157.38 ${\mathrm{cm}}^{-1}$and 1000.41 ${\mathrm{cm}}^{-1}$represent C-O-C and C—O glycosidic bonds typical of polysaccharides. The spectrum for the optimized GCSE sample reveals functional groups linked to bioactive compounds such as polyphenols and alkaloids (Fig. 3b). An absorption band at 3053.44 ${\mathrm{cm}}^{-1}$corresponds to C—H stretching vibrations in cis double bonds (HC=CH) (Aung Moon et al., 2024). O—H stretching between 3500 and 3200 ${\mathrm{cm}}^{-1}$ is indicative of phenolic compounds and alcohols in coffee. Peaks at 2983.05 ${\mathrm{cm}}^{-1}\;$reflect symmetrical and asymmetrical C—H bonds, while the band at 2566.10 ${\mathrm{cm}}^{-1}$represents C—H stretching vibrations. The carbonyl region (1800–1680 ${\mathrm{cm}}^{-1}$) suggests aldehydes, ketones, and esters typical of roasted coffee (Aung Moon et al., 2024).

Absorption bands at 1874.58 *V*${\mathrm{cm}}^{-1}$ represent ester carbonyl groups from triglycerides (O–C=O), while the peak at 1601.23 ${\mathrm{cm}}^{-1}$is associated with caffeine. Peaks at 1335.59 ${\mathrm{cm}}^{-1}$ correspond to C—H bending in trigonelline, and 1233.99 ${\mathrm{cm}}^{-1}$and 1040.88 ${\mathrm{cm}}^{-1}$ relate to chlorogenic acids and coffee fingerprints (Aung Moon et al., 2024). For the optimized microencapsulated formulation (M6), significant intensity changes are observed in the FTIR spectrum (Fig. 3c), indicating interactions between the components. A broad peak at 3386.80 ${\mathrm{cm}}^{-1}$points to hydrogen bonding between the hydroxyl and amine groups of TG-CS and the phenolic groups of the GCSE. The shift in the C=O stretching peak from 1601.23 ${\mathrm{cm}}^{-1}$ to 1693.14 ${\mathrm{cm}}^{-1}$ suggests interactions between polyphenols and chitosan's amine groups. Additional peaks at 1158.45 ${\mathrm{cm}}^{-1}$ and 929.99 ${\mathrm{cm}}^{-1}$confirm the presence of intermolecular hydrogen bonds and potential ionic interactions. These interactions may enhance the stability of the microencapsulated bioactive compounds, causing shifts or broadening of peaks due to hydrogen bonding or ionic interactions between CS, TG, and the phenolic compounds in the GCSE.

#### 3.5.4 XRD

**Fig. 4:**
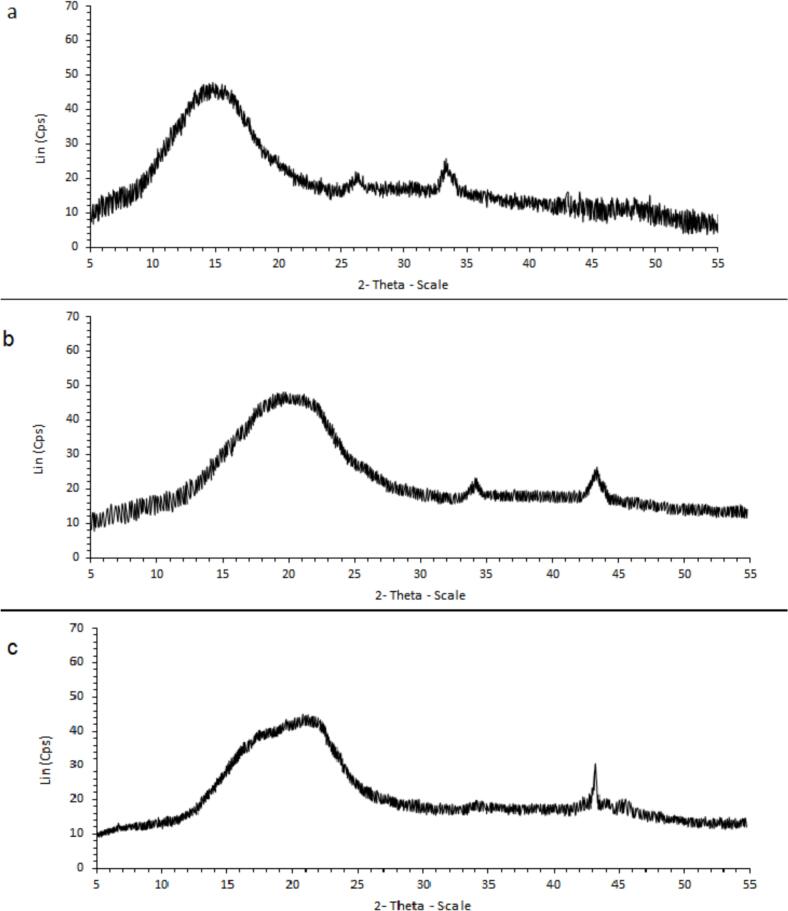
The XRD analysis of (a) TG-CS, (b) Optimized GCSE_8_, and (c) the Optimized microencapsulated (M6).

XRD is a widely used technique for analyzing the structural properties of compounds. Amorphous materials generally exhibit broad, diffuse peaks in XRD patterns, while crystalline substances produce well-defined, sharp peaks. The presence of characteristic peaks can help identify specific compounds. The crystal diffraction patterns, and crystalline structure of biopolymer matrices are shown in Fig. 4a–c, depicting the TG-CS material wall (a), the optimized GCSE sample (b), and the optimized microencapsulated formulation (M6) (c). The XRD pattern of TG-CS material wall (Fig. 4a) exhibits broad, distinct peaks between 10° and 25° at 2θ, indicating an overall amorphous structure for both CS and TG. However, the appearance of a peak around 33° suggests the presence of residual crystalline regions within the CS matrix. The semi-crystalline nature of CS is well-established, with its crystallinity decreasing as the degree of deacetylation increases (El-araby et al., 2022; Karimi et al., 2024).

**Fig. 5:**
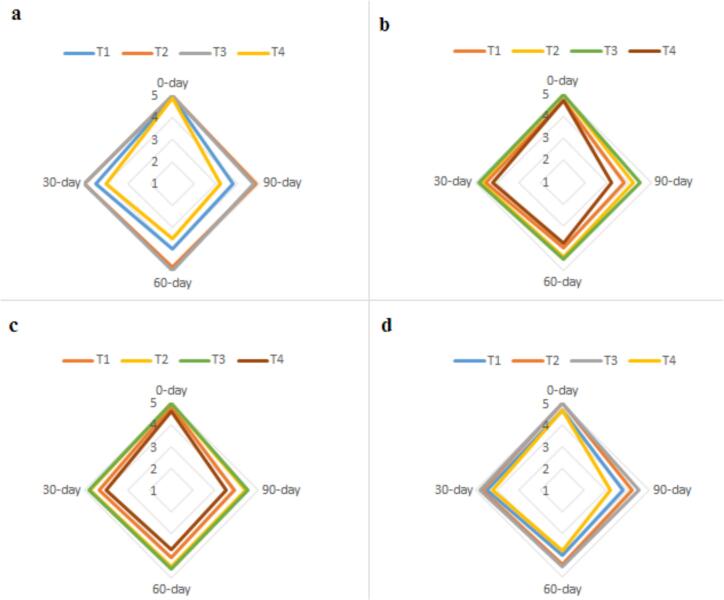
Sensory evaluation results of margarine samples under different treatments (T1: Margarine containing 100 ppm optimized free GCSE_8_, T2: Margarine containing 100 ppm optimized bio-hydrogels encapsulating GCSE_8_, T3: Margarine containing 75 ppm synthetic antioxidant (TBHQ), and T4: Margarine without any additive (Control sample)) over storage periods of 0, 30, 60, and 90th days. Radar plots (a) to (d) correspond to different sensory attributes including (a: Aroma, b: Color, c: Texture, d: Overall acceptance).

Previous studies have linked broad peaks at approximately 20° and 22° with amorphous CS structures (Karimi et al., 2024). The addition of amorphous TG appears to further disrupt the ordered structure of CS, leading to a decline in crystallinity. This is evidenced by the broadening and reduced intensity of diffraction peaks, confirming the formation of a more homogeneous amorphous matrix (Verma et al., 2019). The XRD pattern of GCSE_8_ (Fig. 4b) exhibits both amorphous and crystalline regions. The broad peaks spanning 2θ = 15° to 25° indicate the presence of amorphous components, while crystalline regions appear between 30° and 40°. Hemicellulose polysaccharides are generally known for their amorphous nature (da Silva Júnior et al., 2023). Interestingly, some studies suggest that the thermal processing of coffee beans may contribute to the observed crystallinity within the GCSE_8_ matrix. This transformation is attributed to the removal of water molecules from the crystalline fraction, promoting the conversion of certain α-polymorph structures into the β-crystal phase of caffeine. The XRD pattern of the optimized microencapsulated sample (M6), follows a similar trend to Fig. 5a and b, displaying a broad amorphous region between 2θ = 13° and 25°, which confirms the presence of semi-crystalline structures (Fig. 4c). This is likely due to intermolecular interactions between GCSE and the capsule wall matrix, further supporting the formation of GCSE microcapsules. Additionally, all three patterns exhibit a crystalline region between 30° and 40°. However, Fig. 5c presents a more pronounced peak around 45° compared to the other patterns, which is likely attributed to CS (El-araby et al., 2022).

The increased peak intensity may reflect variations in crystallinity, potentially influenced by thermal conditions or compositional modifications in the M6 sample. Similarly, Mu et al. (2024) analyzed the XRD pattern of microencapsulated green coffee oil (GCO) samples and observed relative crystallinity in the GCO microcapsules. They reported that improved resolution of diffraction peaks in the XRD pattern signifies increased crystallinity, potentially enhancing the structural stability of the microencapsulated sample.

#### 3.5.5 In vitro evaluation of GCSE_8_ release from optimized microencapsulated sample (M6)

To simulate digestive conditions, GCSE_8_ release was monitored for 2 h in SGF and 4 h in SIF, aligning with gastric emptying (112 min) and intestinal transit (120–360 min) times. According to the results presented in Fig. 2S, it is evident that the interaction between the TG-CS carrier and the simulated conditions, facilitating the release of GCSE_8_, was statistically significant (*p* *≤* *0.05*). The release profile of the microencapsulated GCSE_8_ from TG-CS microcarriers, shown in Fig. 2S, demonstrates a steady increase in cumulative release over time. The release rate in SGF was notably higher than in SIF, with 47.38 % of GCSE₈ released during the initial 120 min in SGF. This trend continued in SIF, where the release gradually increased from 120 to 360 min, ultimately reaching 67.33 %. This suggests that the remaining GCSE_8_ may be released over a longer duration in SIF. The faster release in SGF is due to the lower solubility of TG in the acidic conditions, as well as the faster dissolution of CS in this environment, which accelerates microcapsule degradation and GCSE_8_ release (de Siles-Sánchez et al., 2022). The presence of TG may increase the viscosity of the release medium, supporting early release. Additionally, CS and TG's adhesive properties promote interaction with the gastrointestinal mucosa, aiding burst release upon contact (Shirzadian et al., 2021). As the environment transitions from acidic to alkaline pH in the intestine, release kinetics slow down. After 120 min, CS and TG interact with the intestinal fluids, forming a gel-like structure that retains bioactive compounds and results in sustained release. Ionic and hydrogen bonding interactions further strengthen the matrix, reducing dissolution rate, while the pH-sensitive nature of CS slows its solubility in the intestine, contributing to a controlled release (Medha, & Sethi, S., 2024). In this way, the release process is influenced by the different conditions in the stomach and intestine, where initial swelling in the stomach leads to partial release, followed by further disintegration and complete release in the intestinal conditions (Karimi et al., 2024).

### 3.6 Margarine's oxidative stability

#### 3.6.1 Primary oxidation analysis (PV measurement)

Due to its high UFA content, margarine is prone to oxidation, making peroxide value (PV) a key indicator of quality by measuring hydroperoxide formation. As shown in Table 2S, PV was significantly influenced by both time and treatment (*p ≤ 0.05*), with all samples showing increased PV during storage. The control (T4) exhibited the highest increase, from 0.928 ± 0.117 to 7.036 ± 0.123 meq/kg over 90th days (Table 3), while T3 (containing TBHQ) showed the lowest rise (0.927 ± 0.108 to 1.803 ± 0.090 meq/kg). Samples treated with free (T1) and encapsulated (T2) GCSE delayed oxidation effectively, reaching 3.679 ± 0.186 and 2.023 ± 0.066 meq/kg by day 90th, respectively (*p ≤ 0.05*). Initially, free GCSE (T1) showed slightly lower PV than T2 due to immediate antioxidant action. However, from day 60th onward, the encapsulated form outperformed, maintaining PV under 1.244 ± 0.081 meq/kg during the first 60th days and below 2.023 ± 0.066 meq/kg until day 90th. This improved performance is attributed to the protective barrier of the microencapsulation system, which minimizes oxidation and preserves bioactive’ compounds (Al-Asmari et al., 2020). Importantly, all samples remained within the Codex Alimentarius PV limit (≤10 meq/kg) after 90th days, confirming that both free and encapsulated GCSE enhance oxidative stability in margarine (Codex, 1999). This suggests that incorporating both free and encapsulated GCSE_8_ in margarine effectively prevents oxidation and prolongs product stability.

**Table 3:** Oxidative and antioxidant properties of margarine during cold storage.

		No of Treatments	Days			
			0	30	60	90
Oxidative properties	PV* (meq/kg)	T1	0.929 ^aC^ ± 0.051	1.014^bC^ ± 0.119	1.785 ^bB^ ± 0.169	3.679 ^bA^ ± 0.186
		T2	0.929 ^aC^ ± 0.079	1.121^bB^ ± 0.138	1.244 ^cB^ ± 0.081	2.023 ^cA^ ± 0.066
		T3	0.927 ^aD^ ± 0.108	1.097^bC^ ± 0.115	1.269 ^cB^ ± 0.055	1.803^dA^ ± 0.090
		T4	0.928 ^aD^ ± 0.117	2.359^aC^ ± 0.144	3.768 ^aB^ ± 0.152	7.036 ^aA^ ± 0.123
	P-AV* (mMol Aldehyde/kg)	T1	1.341 ^aD^ ± 0.117	2.260^cC^ ± 0.004	4.317^bB^ ± 0.388	8.690 ^bA^ ± 0.426
		T2	1.330 ^aD^ ± 0.078	2.465 ^bC^ ± 0.267	3.0767^cB^ ± 0.199	4.880 ^cA^ ± 0.163
		T3	1.332^aD^ ± 0.138	2.215 ^dC^ ± 0.037	2.856 ^dB^ ± 0.118	4.094 ^dA^ ± 0.197
		T4	1.341^aD^ ± 0.136	4.133 ^aC^ ± 0.218	6.265 ^aB^ ± 0.225	11.143 ^aA^ ± 0.177
	AV* (mg NaOH/g oleic acid)	T1	0.475 ^aD^ ± 0.048	0.583 ^cC^ ± 0.010	1.675 ^bB^ ± 0.115	2.829 ^bA^ ± 0.131
		T2	0.494 ^aD^ ± 0.031	0.634 ^bC^ ± 0.011	1.283 ^cB^ ± 0.075	1.808 ^cA^ ± 0.068
		T3	0.473 ^aD^ ± 0.033	0.551 ^cdC^ ± 0.037	1.128 ^dB^ ± 0.023	1.334 ^dA^ ± 0058
		T4	0.493 ^aD^ ± 0.014	0.899^aC^ ± 0.083	2.766 ^aB^ ± 0.083	4.064 ^aA^ ± 0.053
Antioxidant activity	DPPH* (%)	T1	78.171 ^aA^ ± 0.154	69.026 ^cB^ ± 0.442	58.054 ^cC^ ± 0.614	51.056 ^cD^ ± 0.657
		T2	78.177 ^aB^ ± 0.112	66.349 ^bB^ ± 0.023	64.018 ^bC^ ± 0.318	59.476 ^bD^ ± 0.316
		T3	79.881 ^aA^ ± 0.718	71.862 ^aB^ ± 0.050	78.826 ^aC^ ± 0.173	64.497 ^aD^ ± 0.438
		T4	76.233 ^bA^ ± 0.181	58.002 ^dB^ ± 0.378	48.606 ^dC^ ± 0.362	40.786 ^dD^ ± 0.264

Mean ± SD, *n* = 3. Different small and large superscripts indicate statistically significant differences between the columns and rows, respectively (*p ≤ 0.05*). peroxide value (PV), p-anisidine value (p-AV), Acidity Value (AV) T1: Margarine containing 100 ppm optimized free GCSE_8_, T2: Margarine containing 100 ppm optimized bio-hydrogels encapsulating GCSE_8_, T3: Margarine containing 75 ppm synthetic antioxidant (TBHQ), and T4: Margarine without any additive (Control sample).

#### 3.6.2 Secondary oxidation analysis (p-AV measurement)

The p-anisidine value (p-AV) assesses secondary oxidation by measuring unsaturated aldehydes. As shown in Tables 2S and 3, p-AV increased over time in all samples, mirroring PV trends. After 90th days, the highest p-AV was observed in the control (T4) at 11.143 ± 0.177 mMol/kg, while T3 (with TBHQ) showed the lowest value (4.094 ± 0.197 mMol/kg). T1 and T2 reached 8.690 ± 0.426 and 4.880 ± 0.163 mMol/kg, respectively. All antioxidant treatments significantly reduced secondary oxidation (*p ≤ 0.05*). Although TBHQ's structure (with two para-hydroxyl groups) effectively disrupts oxidation, GCSE—both free and encapsulated—also showed significant effects (*p ≤ 0.05*). Free GCSE (T1) initially outperformed the encapsulated form due to immediate reactivity. However, from day 60th onward, encapsulated GCSE (T2) maintained greater stability, keeping p-AV below 3.076 ± 0.199 mMol/kg for 60th days and under 4.880 ± 0.163 mMol/kg by day 90th. This sustained effect is attributed to the protective microencapsulation matrix and TG-CS complex, which preserve the antioxidant compounds during storage (Karimi et al., 2024; Nateghi & Hosseini, 2023; Santos et al., 2021; Yousefi, Nateghi, & Rashidi, 2024).

#### 3.6.3 Acidity value (*AV*)

AV reflects lipid hydrolysis and free fatty acid (FFA) formation, both of which accelerate oxidation and indicate triacylglycerol breakdown (Santos et al., 2021). According to Table 3, AV increased significantly in all samples during storage (*p ≤ 0.05*), with time × treatment interactions being statistically meaningful (Table 2S) (Yousefi, Hosseini, et al., 2024). By day 90th, the control (T4) showed the highest AV (4.064 ± 0.053 mg NaOH/g), while TBHQ-treated sample (T3) had the lowest (1.334 ± 0.058 mg NaOH/g). Though AV levels in T1 and T2 were similar during the first 30th days, T2 (encapsulated GCSE) showed significantly lower values at days 60th and 90 (*p ≤ 0.05*), underscoring the protective effect of microencapsulation in limiting hydrolytic degradation and preserving antioxidant functionality. The enhanced hydrolytic stability of encapsulated samples was not only due to antioxidant activity but also the barrier properties of the encapsulating matrix. By limiting moisture transfer and enzyme access to triglycerides, the system reduced hydrolysis. Chitosan (CS), with its semi-permeable film-forming nature, restricted water diffusion and shielded oil droplets from hydrolytic agents. Additionally, the compact microcapsule structure lowered enzyme-substrate interaction, further minimizing FFA release (Yousefi, Nateghi, & Rashidi, 2024). TG-CS-based microparticles contributed to FFA stabilization through multiple mechanisms. CS formed a cationic protective layer that repelled pro-oxidant metals, while TG, rich in hydroxyl groups, scavenged free radicals. Moreover, TG-CS hydrogel system created a physical barrier, preserving core bioactive compounds and enhancing structural integrity (Atarian et al., 2019). Collectively, these properties helped suppress hydrolytic degradation and reduce acidity in margarine.

#### 3.6.4 Antioxidant activity assessment

The DPPH scavenging activity of margarine samples was evaluated, and the results are summarized in Table 3, Table 2S. The incorporation of both synthetic and natural antioxidants significantly enhanced DPPH activity in all samples. However, a gradual decline in DPPH activity was observed throughout storage, likely due to the degradation of antioxidants over time (*p ≤ 0.05*). Among the samples, T3 exhibited the highest initial DPPH activity, decreasing from 79.881 ± 0.718 % on day 0 to 64.497 ± 0.438 % by day 90th. In contrast, the control sample (T4) consistently showed the lowest DPPH values, dropping from 76.233 ± 0.181 % to 40.786 ± 0.264 % over the storage period. Although no antioxidant was added to the control sample (T4), its initial DPPH activity can be attributed to naturally occurring antioxidants present in the base formulation. Vegetable oils commonly used in margarine, such as canola, sunflower, and palm oils, inherently contain tocopherols with well-documented radical scavenging capacity. Furthermore, soy lecithin included in the formulation may also exert mild antioxidant effects due to its phospholipid content. These intrinsic components likely account for the relatively high DPPH value of the control at day 0 (Yousefi, Hosseini, et al., 2024). Samples T1 (78.171 ± 0.154 % to 51.056 ± 0.657 %) and T2 (78.177 ± 0.112 % to 59.476 ± 0.316 %) demonstrated higher antioxidant activity than T4, though their values remained lower than T3. Notably, a distinct difference emerged between the free and encapsulated forms of GCSE during storage. In the early phase (up to day 30th), the free-form GCSE exhibited strong DPPH activity, indicating its initial antioxidant potential. However, as storage progressed, its effectiveness declined significantly, likely due to chemical degradation and reduced radical-scavenging capacity caused by exposure to oxidation-promoting factors. Conversely, the encapsulation technique provided enhanced protection against degradation, leading to improved stability of the incorporated antioxidants. This effect became particularly evident in T2, where DPPH activity was better preserved by day 90th compared to the free-form GCSE (T1). Several studies have highlighted encapsulation as an effective method for prolonging antioxidant efficacy by shielding bioactive compounds from oxidation (Karimi et al., 2024; Yousefi, Nateghi, & Rashidi, 2024). Additionally, the positively charged GS structure contributed to oxidative stability, further supporting the long-term preservation of antioxidant activity in the encapsulated samples (Atarian et al., 2019).

#### 3.6.5 Sensory evaluation

Sensory evaluation plays a crucial role in determining consumer acceptability and preference. To assess the impact of TG-CS coated microparticles of GCSE in margarine, sensory attributes were analyzed over a 90-day of cold storage (Fig. 5). The results showed a consistent trend across all treatments, with T1 and T4 exhibiting significant declines in sensory properties (*p > 0.05*), indicating oxidative degradation and structural breakdown. In contrast, T2 and T3 maintained better stability, confirming the protective effect of microencapsulation and TBHQ, in alignment with PV and p-AV measurements. Among all treatments, T3 demonstrated the highest stability, followed closely by T2, while T1 and T4 showed substantial deterioration, with T4 receiving the lowest scores at 90th days. The addition of GCSE in encapsulated form had no negative effects on margarine, as it protected the product from oxidative factors, preserving its ability to act as an antioxidant. These findings align with previous studies indicating that antioxidant-free margarine undergoes faster oxidative and sensory degradation, leading to reduced consumer acceptability (Ouahrani et al., 2022).

## 4 Conclusion

This study successfully encapsulated green coffee antioxidants within chitosan-tragacanth (TG-CS) hydrogels, demonstrating enhanced physicochemical stability and antioxidant performance compared to free antioxidants. The optimal TG-CS formulation achieved efficient encapsulation, with SEM, FTIR and XRD analyses confirming the structural integrity and successful entrapment of bioactive compounds. In vitro release studies under simulated digestive conditions revealed sustained and controlled release of antioxidants, suggesting their potential for targeted delivery in lipid-rich systems.

When applied to margarine, the encapsulated antioxidants effectively mitigated oxidative degradation while preserving key quality parameters such as aroma, texture, and color. Sensory evaluation indicated strong consumer acceptance, highlighting the formulation's compatibility with food matrices. The findings underscore the viability of TG-CS microencapsulation as a delivery system for natural antioxidants, aligning with clean-label trends and functional food innovation.

These results hold promises for broader applications in lipid-based products, including baked goods and plant-based alternatives, to enhance shelf life and nutritional value. Future research should explore higher antioxidant concentrations, synergies with natural stabilizers, and scalability for industrial applications. This work bridges natural bioactive potential with advanced delivery technologies, offering sustainable solutions for food preservation.

## CRediT authorship contribution statement

**Masoumeh Javadpour:** Investigation, Formal analysis. **Elahesadat Hosseini:** Writing – review & editing, Writing – original draft, Software, Investigation, Formal analysis. **Leila Nateghi:** Supervision, Data curation, Conceptualization. **Sara Bazrafshan:** Writing – review & editing, Writing – original draft, Investigation, Data curation.

## Funding

The authors confirm that this research was conducted without any financial support or grant funding from external sources.

## Declaration of competing interest

The authors declare that they have no known competing financial interests or personal relationships that could have appeared to influence the work reported in this paper.

## Data Availability

Data will be made available on request.

## Appendix A Supplementary data

Supplementary material Image 1
