# Supplementary material for: Enhancing margarine oxidative stability, antioxidant retention, and sensory quality via tragacanth-chitosan hydrogel microencapsulation of supercritical CO₂-extracted green coffee
Source: Food Chem X. 2025 May 23;28:102580. doi: 10.1016/j.fochx.2025.102580 (PMC12152904; doi:10.1016/j.fochx.2025.102580)
Supplement: Supplementary file 1 — Supplementary material [file mmc1.docx]

**Supplementary Material**

**Table1S. Fatty acids composition of** optimized GCSE_8_

| Peak No | Fatty acid | Retention time (min) | Concentration (%) |
| --- | --- | --- | --- |
| 1 | Myristic acid (C14:0) | 8.45 | 3.564 ± 0.05 |
| 2 | Palmitic acid (C16:0) | 10.05 | 22.499 ± 0.44 |
| 3 | Oleic acid (C18:1) | 14.05 | 6.065 ± 0.13 |
| 4 | Linoleic acid (C18:2) | 15.14 | 50.239 ± 0.39 |
| 5 | Stearic acid (C18:0) | 16.11 | 6.734 ± 0.09 |
| 6 | Arachidic acid (C20:0) | 17.35 | 3.043 ± 0.06 |
| 7 | Behenic acid (C22:0) | 19.62 | 3.622 ± 0.03 |
| 8 | Lignoceric acid (C24:0) | 21.33 | 4.233 ± 0.08 |


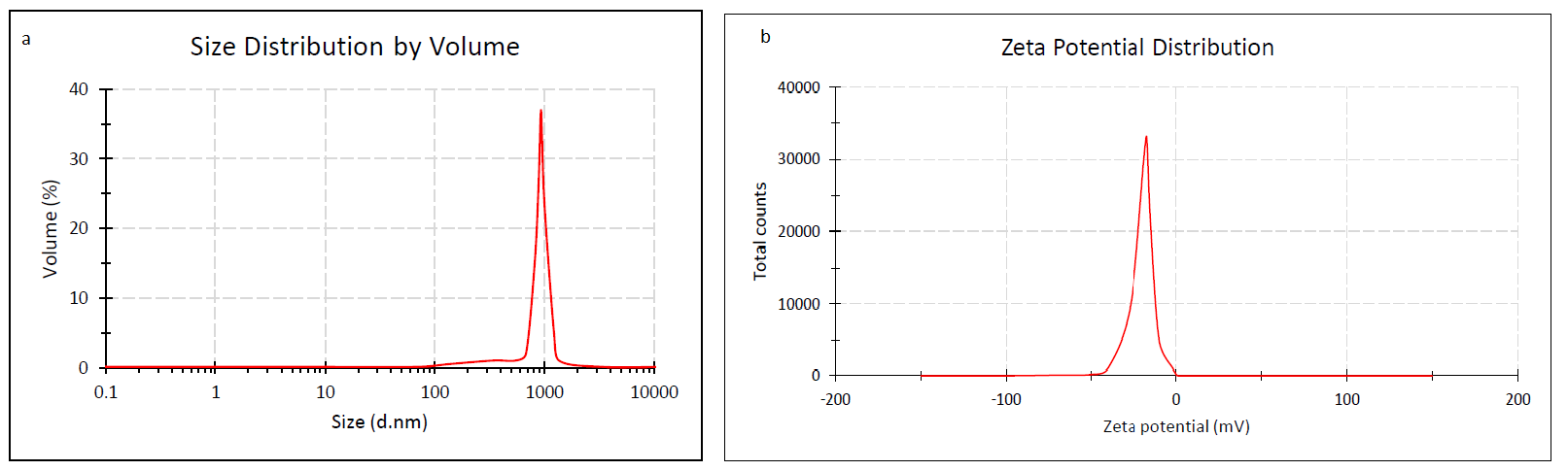


**Figure 1S**. Particle size distribution (a), Zeta potential distribution (b) of Optimized Microencapsulated

**Figure 2S**. *In vitro* release profile of the microencapsulated sample (M6) in simulated gastric fluid (SGF) and simulated intestinal fluid (SIF).

**Table 2S**. ANOVA for the characteristics of margarine

| Source of validation | Oxidative properties | | | | | | Antioxidant activity | |
| --- | --- | --- | --- | --- | --- | --- | --- | --- |
|  | PV | | p-AV | | AV | | DPPH | |
|  | P-value | F-value | P-value | F-value | P-value | F-value | P-value | F-value |
| Storage time (A) | 0.00^*^ | 1326.92 | 0.00^*^ | 1708.50 | 0.00^*^ | 2675.45 | 0.00^*^ | 9095.6 |
| Type of margarine (B) | 0.00^*^ | 2775.24 | 0.00^*^ | 1278.04 | 0.00^*^ | 890.98 | 0.00^*^ | 3759.63 |
| Interaction effect (A×B) | 0.00^*^ | 254.87 | 0.00^*^ | 139.55 | 0.00^*^ | 232.57 | 0.00^*^ | 287.34 |
| Source of validation | Sensory evaluation | | | | | | | |
|  | flavor | | color | | texture | | Overall acceptance | |
|  | P-value | F-value | P-value | F-value | P-value | F-value | P-value | F-value |
| Storage time (A) | 0.007^*^ | 5.75 | 0.119 | 2.28 | 0.021^*^ | 4.29 | 0.001^*^ | 9.63 |
| Type of margarine (B) | 0.001^*^ | 9.11 | 0.219 | 7.79 | 0.081 | 2.69 | 0.001* | 10.32 |
| Interaction effect (A×B) | 0.011^*^ | 0.95 | 0.114 | 7.06 | 0.990 | 0.20 | 0.895 | 0.44 |

*Indicates significance at the 5% level (*p < 0.05*)
